# Does a Combination of Virtual Reality, Neuromodulation and Neuroimaging Provide a Comprehensive Platform for Neurorehabilitation? – A Narrative Review of the Literature

**DOI:** 10.3389/fnhum.2016.00284

**Published:** 2016-06-24

**Authors:** Wei-Peng Teo, Makii Muthalib, Sami Yamin, Ashlee M. Hendy, Kelly Bramstedt, Eleftheria Kotsopoulos, Stephane Perrey, Hasan Ayaz

**Affiliations:** ^1^Institute for Physical Activity and Nutrition (IPAN), Deakin University, BurwoodVIC, Australia; ^2^EuroMov, University of MontpellierMontpellier, France; ^3^Cognitive Neuroscience Unit, Deakin University, BurwoodVIC, Australia; ^4^Liminal Pty Ltd., MelbourneVIC, Australia; ^5^Adult Mental Health, Monash Health, DandenongVIC, Australia; ^6^School of Exercise and Nutrition Sciences, Deakin University, BurwoodVIC, Australia; ^7^Aged Persons Mental Health Service, Monash Health, CheltenhamVIC, Australia; ^8^School of Biomedical Engineering, Science and Health Systems, Drexel University, PhiladelphiaPA, USA; ^9^Department of Family and Community Health, University of Pennsylvania, PhiladelphiaPA, USA; ^10^The Division of General Pediatrics, Children’s Hospital of Philadelphia, PhiladelphiaPA, USA

**Keywords:** neurorehabilitation, neuroplasticity, tDCS, fNIRS, EEG, virtual reality therapy

## Abstract

In the last decade, virtual reality (VR) training has been used extensively in video games and military training to provide a sense of realism and environmental interaction to its users. More recently, VR training has been explored as a possible adjunct therapy for people with motor and mental health dysfunctions. The concept underlying VR therapy as a treatment for motor and cognitive dysfunction is to improve neuroplasticity of the brain by engaging users in multisensory training. In this review, we discuss the theoretical framework underlying the use of VR as a therapeutic intervention for neurorehabilitation and provide evidence for its use in treating motor and mental disorders such as cerebral palsy, Parkinson’s disease, stroke, schizophrenia, anxiety disorders, and other related clinical areas. While this review provides some insights into the efficacy of VR in clinical rehabilitation and its complimentary use with neuroimaging (e.g., fNIRS and EEG) and neuromodulation (e.g., tDCS and rTMS), more research is needed to understand how different clinical conditions are affected by VR therapies (e.g., stimulus presentation, interactivity, control and types of VR). Future studies should consider large, longitudinal randomized controlled trials to determine the true potential of VR therapies in various clinical populations.

## Introduction

**FIGURE 1:**
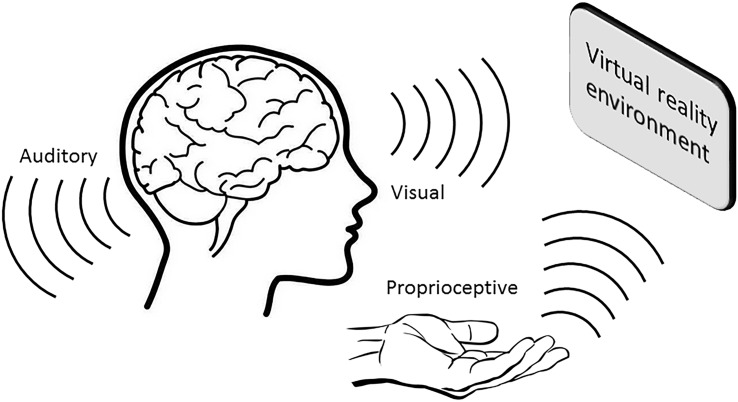
**A VR environment induces multisensory feedback that contributes toward greater memory consolidation and retention**.

In the last two decades, the application of VR training has become increasingly popular, not only as a means to enhance gaming experiences, but also in the education and healthcare settings to improve learning and rehabilitation outcomes. Particularly in the area of neurorehabilitation, the use of VR technology has shown great promise by providing a sense of realism during training, thereby promoting skill acquisition and retention, and inducing functional recovery (**Figure 1**; for review, see Adamovich et al., 2009).

In the context of neurorehabilitation, VR therapy can be described as a method of brain–computer interaction that involves real-time simulation of an environment, scenario or activity that allows for user interaction and targets multiple senses. In particular, the combination of VR and recent technological advances in robotic and haptic interfaces allow users a seemingly life-like interactional experience in a VE (Jung et al., 2012; Yeh et al., 2014). For example, VR has been used in clinical settings as a training tool for surgeons to learn intricate fine motor skills associated with precision surgery (Wang, 2012; Fang et al., 2014), and as a tool to deliver cognitive-based therapies (Kim et al., 2011; Kandalaft et al., 2013). More complex forms of VR presentation such as augmented VR (whereby VR is superimposed on the actual environment) and immersive VR (first-person interaction in a VR environment) brings the immersive experience to another level with technology such as head-mounted displays (i.e., Oculus^®^ Rift and Microsoft^®^ Hololens) or screens. It is through this naturalistic environment, and allowing for interactive behaviors while being monitored and recorded, that is the primary advantage of implementing VR technology. This means that VR technology can be used to deliver meaningful and relevant stimulation to an individual’s nervous system and thereby capitalize on neuroplasticity to promote both cognitive and motor rehabilitation.

In this review, we will discuss the theoretical framework for the use of VR in the context of neurorehabilitation. We will provide evidence for the use of VR in motor rehabilitation for neurological disorders such as PD, CP and stroke and in mood and mental health disorders such as anxiety, PTSD and schizophrenia. We will also review the concurrent use of non-invasive brain stimulation and neuroimaging techniques during VR, discussing how these combined techniques may augment the benefits and complement current VR training protocols.

## Theoretical Framework For VR and Learning

### Experiential Learning

The most important aspect of using VR is to provide new experiences by allowing users to interact physically and emotionally within a VE that is almost identical to the real world. The combination of physical, mental and emotional interaction encourages active participation and involvement of the user. In this sense, users of VR assimilate knowledge more effectively when they have the freedom to engage in self-directed activities within their learning context. By finding solutions and learning new skills autonomously, users of VR invest mental effort by constructing conceptual models that are both consistent with what they already understand and with the new content that is presented (Garrison and Garrison, 1997). Another key feature of VR training is that it offers users the opportunity to acquire skills in the context where they need to be applied. This results in more meaningful and effective learning, as compared with learning out of context (Nieuwenhuijsen et al., 2006). In physical rehabilitation for example, rehabilitation of fine motor control of the hands and wrists can be “re-trained” by simulating a VE where a stroke patient needs to pour him or herself a glass of water in the kitchen. In this way, patients practice and refine fine motor control of muscles controlling the hands and wrists through manipulating a virtual object that allows the same kind of natural interaction with objects that patients would engage in the real world.

### Augmented Feedback: Knowledge of Results and Performance

Another important aspect of VR therapy is the ability to provide augmented feedback to its users. Augmented feedback is additional information provided through any means (e.g., visual, auditory or kinesthetic) that is complimentary to the inherent feedback received via the sensory systems. There is no hard and fast rule as to how or what kinds of information augmented feedback should provide, however, VR therapy offers two vital pieces of information that is essential for learning (Winstein, 1991; Lauber and Keller, 2014); (1) knowledge of performance – information on how the participant performs during movement (i.e., movement sequences, joint angles, force outputs at each phase of movement etc.); (2) knowledge of results – information on the outcome of the performance (i.e., overall quality and quantity of movement). Currently, most commercially available VR games would incorporate visual, auditory and even kinesthetic feedback that can be provided either during or after the game. Very often, these VR games are designed in a manner that users have to maintain or achieve a pre-determined score or level in order for the game to progress. For example, VR applications can provide knowledge of performance throughout gameplay in the form of movement kinematics (i.e., joint angles, velocity, and speed), kinetics (i.e., ground reaction forces and torque) or even the level of activation in specific brain regions during a particular task. In order for users to progress to the next level, users must maintain or exceed a threshold that has been set based on previous trials or specific performance outcomes. Upon task completion, knowledge of results and performance can be provided, allowing both clinicians and users to understand deficiencies in movement patterns that are associated with specific dysfunctional movement outcomes, apply progressions appropriately, and address those deficiencies with a targeted rehabilitation approach.

### Observational Learning

Apart from providing feedback that is necessary for learning, another aspect of VR training is the enhancement of observational learning. The basis for learning, or at least its intended outcome, is to mimic or replicate an ideal response that brings about a desired result and induce a lasting change in behavior. In terms of neurorehabilitation, observation of goal-oriented movements or processes provides sensory feedback about the movement, behavior or emotional state, which contributes to learning (Oouchida et al., 2013; Williams and Carnahan, 2014). These observations preferentially activate parts of the brain that are involved with the physical performance itself allowing a motor program to be developed based upon the observed movements (Burke et al., 2010). Training in a VE may facilitate observational learning in four different ways; (1) VR applications can provide an accurate visual representation of the user’s body and limb position using motion capture technology; (2) VR applications commonly use an avatar to mimic the movement of users, or conversely, the user could mimic the movements of the avatar; (3) accurate guides or a correct movement pattern can be produced for which users can follow; (4) VR applications can facilitate mental imagery by inducing optimal mood states and instructions for mental imagery.

### Motivation

Importantly, the goal-oriented nature of VR tasks may support the maintenance and adherence of neurorehabilitation programs. Unlike traditional therapist-led sessions, where improvements in physical or cognitive function may be subjective or difficult for patients to identify (Van den Broek, 2005), VR programs can provide an objective, quantitative measure of session outcomes and objectives. Furthermore, VR applications can provide both users and clinicians the ability to individualize training programs or alter the progression of a training session based upon the user’s personal performance. The capacity to individualize therapy intensity may enhance motivation by allowing users to select practice sessions that are catered to their individual time and need, and more importantly, to manipulate treatment parameters to create optimal learning conditions. Another important consideration for VR to improve motivation is by incorporating competition or co-operation between other players during therapy sessions. Engaging users in a group environment either competing against each other or working in teams promotes an element of enjoyment through increased social interaction, particularly amongst people suffering with similar conditions (Van den Broek, 2005).

## Evidence of VR Therapy in Movement Neurorehabilitation

### Stroke

The use of motion-controlled VR game consoles, including the Nintendo^®^ Wii and Xbox^®^ Kinect, have been explored as adjuncts to conventional physical therapy (see **Table 1**), specifically for improving upper limb function (Thomson et al., 2014; Laver et al., 2015). VR programs for stroke neurorehabilitation are based on the potential for brain neuroplasticity after neurological injury to support acquisition and retention of new motor skills to recover motor function. The goal of VR therapy in stroke is to apply these motor learning principles for stroke neurorehabilitation, such as providing repetitive, graded intensity, and motivating task-specific training with real time multimodal feedback of movements and performance (Saposnik et al., 2011). Thus, VR systems are designed to enhance conventional therapy by providing a tool to deliver more specific, intensive and enjoyable therapy with real time feedback of performance (Levin et al., 2015).

**Table 1:** Examples of recent systematic reviews and meta-analyses demonstrating the effects of VR in neurorehabilitation of stroke, PD and CP.

Author and year	Study aims	Studies included and sample (*n*)	Study outcomes	Points of discussion
**Stroke**				
Laver et al. (2015)	Compared the effects of virtual reality on arm function, walking speed and independence in managing daily activities after stroke versus an alternative intervention or no intervention.	37 studies (*n* = 1019)	12 studies found improved arm function.	Low sample size in most studies.
			4 studies found improved walking speed.	Some studies reported pain, headaches or dizziness in small number of participants, but no adverse events overall.
			8 studies found slight improvements in activities of daily living.	Low quality evidence for arm function.
				Very low quality evidence for walking ability, global motor function and independence in performing daily activities.
				The quality of the evidence for each outcome was limited due to small numbers of study participants, inconsistent results across studies and poor reporting of study details.

Corbetta et al. (2015)	Compared the effects of VR-based rehabilitation on gait, balance and mobility versus standard therapy.	15 studies (*n* = 341)	Significant improvements in walking speed, balance, and mobility.	Substituting some or all of a standard rehabilitation regimen with VR training provides greater benefits in walking speed, balance, and mobility.
			Significant improvements in mobility if VR training was combined with standard therapy.	Although the benefits are small, the cost of administering VR is also small particularly when patient demand is high in a clinic setting.
			Insufficient evidence to support to use of combined VR and standard therapy on balance and walking speed.	

Luque-Moreno et al. (2015)	Compared the effects of VR interventions on lower extremity rehabilitation.	11 studies (*n* = 231)	High heterogeneity in study designs.	VR interventions (more than 10 sessions) may have a positive impact on lower limb function.
			Small sample sizes. Mean sample size of 20 per study.	Multimodal approach (i.e., a combination of VR and conventional therapy) may elicit greater results.
			Studies were ranked between 4 and 7 points (out of 10) on the PEDro scale.	Adaptability of software seemed to adapt better to patient’s requirements, allowing for individualized treatments.

Lohse et al. (2014)	Compared the effects of custom built virtual games and commercially available gaming systems.	26 studies (*n* = ?)	Only 4 studies used commercial games while 20 studies used custom built virtual games.	VR intervention improves outcomes compared to conventional therapies.
			Mean PEDro score for all studies was 5.42 ± 1.6 (out of 10).	Small samples and few number of studies in commercial games limits the assessment of potential benefits.
			Methodological limitations of studies include subject, experimenter and therapist blinding, small sample size, and difficulty in determining a dose-response effect.	
			Significant improvements in body function and activity outcomes.	

**Parkinson’s disease**				
Harris et al. (2015)	Compared the effects of exergaming on static and dynamic balance in older adults and PD.	11 studies (*n* = 325 healthy older adults, 56 PD)	9 studies showed a significant improvement in static balance and postural control in healthy aging individuals.	Few studies in PD and small sample size limits the interpretation of the effectiveness of exergaming in PD.
			2 studies found a significant improvement in static balance and postural control people with PD.	Evidence found in this meta-analysis supports the use of exergaming as an adjunctive tool to improve balance and postural control.
			Studies were ranked between 4 and 8 points (out of 10) on the PEDro scale.	

Barry et al. (2014)	Examined the safety, feasibility and effectiveness of exergaming in people with PD.	7 studies (*n* = 110)	Only 2 studies addressed patient safety. No objective measures (such as falls or near falls) or subjective measures (patient’s perception) were recorded in any studies.	While the effectiveness and feasibility are often measured, more research is required to establish the safety, particularly in home-based VR therapy.
			Only 1 study recorded gameplay experience. Good levels of motivation during game play were reported although difficulties with the fast pace and cognitive complexity of some games were raised.	The use of commercial games may be too difficult for some people with PD, and exergames that tailor specifically to the needs and capabilities of patients may be more effective.
			Exergaming was found to be just as effective as standard physical therapy for improving clinical measures of balance and cognition even up to 60 days post-intervention.	

**Cerebral Palsy**				
Dewar et al. (2015)	Systematic review of various interventions to improve postural control in children with CP	45 studies (*n* = ?)	4 studies investigated the use of VR on postural control.	The systematic review provided conflicting evidence of VR on postural control and gait.
			2 studies were rated weak in study conduct while 2 had a strong study design.	Due to the preliminary nature of these studies, it is difficult to truly ascertain if indeed the use of VR had any effects on postural control and gait.
			3 studies showed improvements in balance, while 2 study showed improvements in walking capacity.	

Chen et al. (2014)	Examined the effects of virtual gaming on upper extremity function in children with CP	14 studies (*n* = 122)	3 RCTs, 2 cohort studies, 7 case studies and 2 single-subject design studies.	The use of VR may be highly applicable in a pediatric population.
			For 3 RCTs, no difference was found between VR therapy and conventional therapy.	Small sample size and the lack of large RCT is a limiting factor in interpreting the results.
			Overall upper extremity function was significantly improved after VR therapy.	
			Strongest effects of VR was shown in younger children, custom-built systems in the home or laboratory setting.	


*PEDro, physiotherapy evidence database (PEDro); RCT, Randomized controlled trials; PD, parkinson’s disease; CP, cerebral palsy; VR, virtual reality.*

Despite the potential utility of commercial VR game consoles for stroke neurorehabilitation, a number of limitations have been highlighted (Bower et al., 2015): (1) VR games designed for the general population can be too challenging for stroke patients with physical and cognitive deficits; (2) the difficulty levels and control of VR games are often not readily adjustable to rehabilitation targets, and the tasks may lack functional relevance; (3) feedback and scoring provided can be negative and frustrating for the user; (4) current VR games do not include neurological assessment; (5) VR does not integrate multiple environmental factors that connect to motor performance. In response to some of these limitations, there has been an emergence of research and development of modified VR programs specifically designed for stroke neurorehabilitation using adaptable software and hardware components of commercial VR systems (e.g., Kinect system) and guidance from clinicians in their development (Laffont et al., 2014; Bower et al., 2015). These adapted VR systems are progressively optimized with new functions including: (1) allowing automatic adaptation/intensity grading of the activity to the patient’s own achievements; (2) allowing the therapist to adapt online the task’s characteristics to the patient’s needs; (3) allowing multiplayer VR systems via a web-service platform to enhance interactivity; (4) automatic recording of the patient’s movements to provide therapists with data describing the quality and quantity of motor function recovery/progression, including the level of compensatory movements (Laffont et al., 2014).

While there is some evidence to suggest that VR may be highly applicable for stroke rehabilitation, the evidence from recent systematic reviews and meta-analyses indicate that current studies are limited by sample size issues and study designs (see **Table 1**). Early VR interventions used commercial applications such as the Nintendo Wii that controls an avatar, however, more recently customized systems have focused on interactive platforms to target activities of everyday living (i.e., reaching and grasping tasks). However, a major challenge with stroke is that no one stroke patient will present with the same motor deficit and therefore an individualized approach to therapy, including VR therapy, is needed. In this sense, future systems must be adaptive and customizable to manage the heterogenous nature of stroke for patients to gain greater benefits.

### Parkinson’s Disease

Emerging VR therapies presents as an attractive option for delivering neurorehabilitation therapies to manage the cognitive-motor symptoms in people with PD, as it can be employed at any stage as an adjunct to standard pharmacological (Levodopa therapy) and/or surgical (ablation, deep brain stimulation) treatment (see **Table 1**). This new possibility in the field of neurorehabilitation aims to provide PD patients with a motivating way to perform multiple motor neurorehabilitation exercises with the rationale that the VR system might promote balance training, and cognitive-motor practice. Some commercial VR systems, such as the Nintendo^®^ Wii system using a balance board, has drawn considerable attention from both the research and clinical communities as effective and feasible neurorehabilitation interventions to enhance gait and balance for people with PD (Barry et al., 2014; Harris et al., 2015). More recent studies have implemented custom programming and hardware to their VR systems to specifically improve balance and gait in PD (Mirelman et al., 2011).

It has been demonstrated that a VR neurorehabilitation program of 6–8 weeks involving 40–60 min a day, three times per week appears to be a viable option for significantly improving balance in a clinical population of individuals with PD (Esculier et al., 2012). The intensity/difficulty load of interventions used across existing studies appears a key contributing factor for the discordant findings reported in the literature (Esculier et al., 2012). In addition, activity selection could have contributed to some differential findings among studies. Some studies targeted static slow controlled movements in a closed environment such as the Wii Fit with balance board (dos Santos Mendes et al., 2012; Esculier et al., 2012), while others involve dynamic movements in an open environment such as Wii Sports (Herz et al., 2013).

Despite some evidence for performance improvement in balance, there are still limitations inherent in commercial VR systems that may not directly apply to realistic everyday settings for PD neurorehabilitation. Additionally, the programs are not very scalable, or modifiable, to each individual’s needs or progress for all stages of disease. Mirelman et al. (2011, 2013) utilized a custom-made VR system to incorporate virtual obstacles presented on a screen during treadmill walking (18 sessions over 6 weeks). During the gait training they used a novel method (V-TIME) for tracking foot position based on the X-box Kinect technology. Interestingly, Mirelman et al. (2011) observed significant elevated gait speed with and without a cognitive dual-task upon completion of training and 4 weeks post-training. However, this VR gait training protocol confines participants to straight-walking, a gait pattern that is relatively uncommon in real-life environments. Perhaps a more viable approach may be the development of a VR system that may be used in conjunction with activities of daily living. People with PD are known to use visual and/or auditory cues to improve physical performance (Lee et al., 2012), and perhaps the use of augmented VR, via goggles or smart glasses, may be used to provide sensory cues as a feedforward or feedback mechanism to improve physical performance.

### Cerebral Palsy

Cerebral Palsy (CP) is the most common pediatric physical disability, thought to affect three to four individuals per 1000 of the population (Aisen et al., 2011; Oskoui et al., 2013) characterized as a spectrum of disorders of motor and postural development that cause limited functionality or dysfunction (Monge Pereira et al., 2015). Studies investigating exercise-based treatments for children with CP has provided growing evidence in the last decades for effectiveness in improving postural control (see **Table 1**). Although effective, traditional physical exercise in the clinical settings consists of repetitive tasks that limits the enthusiasm over regular periodic application.

While the study of VR in children with CP is still at its infancy, Denise Reid at the University of Toronto’s Virtual Reality Laboratory (Reid, 2002a,b, 2004) has provided preliminary evidence to support its use. In these studies, children with CP were engaged with VR based exercises for upper extremity and postural control. The self-reported effect of VR on perceived self-efficacy to perform given tasks was tested in an uncontrolled study, before and after intervention (Reid, 2002a). Based on the self-efficacy theory, Reid (2002b) attempted to identify if use of VR could increase the motivation for exercise in children with CP. The pilot study yielded encouraging results for VR use with improvement in perceived performance abilities and satisfaction with performance. In a follow up study on upper-extremity efficiency, improvement was also reported with VR use (Reid, 2002b). Similarly, Reid (2004) later investigated the effect of VR intervention on playfulness and found that VR environments stimulated playfulness in children, specifically the VR tasks that allowed creativity, expression, and choice of activity. You et al. (2005) used fMRI in a case report to investigate cortical reorganization and associated motor function improvement after a VR therapy. Neuroplastic changes were observed in the primary sensorimotor cortex and supplementary motor area following VR therapy, together with enhanced functional motor skills. A later study by Bryanton et al. (2006) compared the VR therapy with conventional exercises in children and found that although children completed more repetitions of the conventional exercises, the range of motion and hold time in stretched position was greater during VR tasks.

While the current research for VR in children is still in the early stages, VR therapies represent a viable option to increase exercise adherence and physical activity as they are both engaging and rewarding particularly in an adolescent population. The process of gamification, one that entails an interactive dynamic storyline and an overall goal, is likely to better capture and retain the attention of children over traditional physical training (Lister et al., 2014). The challenge, particularly in children with CP, will be to incorporate a diversity of activities performed during the game to train a repertoire of fundamental skills so as to further develop their motor and cognitive skills.

## Evidence For VR Therapy in Cognitive Rehabilitation and Mental Health

### Anxiety, Phobias and Post-traumatic Stress Disorder

Anxiety can be generalized in nature [i.e., generalized anxiety disorder (GAD)], characterized by long-lasting anxiety that is not focused on a specific object, or may be more focal (i.e., phobias) occurring in the presence of, or in anticipation of, a specific object or situation. Preliminary evidence on the use of VR in GAD indicate that a combination of relaxation, controlled exposure and stress inoculation may help patients to cope with various stressors and sources of worry (Gorini et al., 2010; Repetto and Riva, 2011). Additionally, the combination of biofeedback (e.g., heart rate and electro-dermal skin response) may potentially help to identify particular sources of worry and emotion that can be used to modify specific features of the VR environment (Gorini et al., 2010; Repetto and Riva, 2011).

Despite the limited evidence for the use of VR therapy in GAD, there is some support for the use of VR in a range of other anxiety disorders (see **Table 2**) including specific phobias (Cote and Bouchard, 2005; Maskey et al., 2014), panic disorder (Vincelli et al., 2002) and social phobia (Klinger et al., 2004, 2005). Current VR therapies, particularly for phobias, use controlled exposure therapy that allows the patient to experience a sense of presence in an immersive, interactive VE that minimizes avoidance behavior and facilitates emotional involvement. This VE also allows controlled delivery of sensory stimulation via the therapist, for which the patient confronts the feared stimuli in a progressive manner. Another advantage of VR therapy is being able to recreate situations that cannot be re-experienced *in vivo* (i.e., combat situation or terrorist attack). VR therapy may be used as an alternative to imaginal exposure, meaning that patients with PTSD need not rely on internal imagery to visualize an event. A potential limitation in imaginal exposure therapy is that the therapist has no control over, or even knowledge of, what imagery the patient actually evokes (Strosahl and Ascough, 1981). Whereas in the VE, the stimuli presented can be carefully controlled and monitored. As with phobic patients, VR-based exposure therapy may be particularly useful for patients with PTSD for whom avoidance and failure to engage with therapy may hinder the therapeutic process. The efficacy of VR therapy for the treatment of PTSD has predominantly been examined in military populations (Cukor et al., 2009; Goncalves et al., 2012). A systematic review found that VR therapy was just as efficacious as traditional exposure treatment for PTSD (Goncalves et al., 2012). Seven of the 10 studies included in Goncalves’s review found that VR environments significantly reduced PTSD symptoms in comparison to control, however, no significant differences in symptoms were observed between VR therapy and traditional exposure treatment.

**Table 2:** Examples of systematic reviews and meta-analyses demonstrated the use of VR in treating PTSD and anxiety disorders.

Author and year	Study aims	Studies included and sample (n)	Study outcomes	Points of discussion
**PTSD**				
Kuester et al. (2016)	Examined the efficacy of internet-based CBT and expressive writing in people with PTSD vs. waitlist or active controls.	20 studies (*n* = 973 intervention, 805 controls)	Internet-based CBT are showed medium to large effect sizes compared to passive controls, but not against active controls receiving face-to-face CBT with therapist.	Internet-based CBT may be just as beneficial as conventional CBT.
				Due to large variability in outcome measures of included studies, subgroup analyses was limited.

Goncalves et al. (2012)	A systematic review of the efficacy of VR exposure therapy in the treatment of PTSD vs. waitlist or active controls.	10 studies (*n* = ?)	Patients in VR exposure therapy showed insignificantly better results compared to waitlist controls, but no differences was observed when compared to exposure therapy.	Preliminary evidence suggests that VR exposure therapy is just as efficacious as conventional CBT.
			Majority of VR exposure therapy used head-mounted displays and customized virtual environment specific to the condition.	Studies included did not use intent-to-treat analysis or did not state concomitant treatments and/or comorbidities.
			No difference in dropout rates between VR therapy and conventional CBT.	

Anxiety disorder				
Opris et al. (2012)	Treatment efficacy of VR exposure therapy vs. conventional CBT in anxiety disorders vs. active controls.	23 studies (*n* = 608)	VR therapy was significantly better than waitlist controls.	The use of VR exposure therapy may be a viable option.
			Similar improvements were observed between VR and conventional CBT therapy.	There is a future need to determine the use of VR exposure therapy to other forms of VR therapies targeted at anxiety disorders.
			Similar improvements in outcome measures were maintained over time in both the VR and conventional CBT groups.	No measure of dropout rates in studies reviewed.

Powers and Emmelkamp (2008)	Examined the effects of VR in anxiety disorders vs. waitlist or conventional CBT controls.	13 studies (*n* = 397)	VR therapy was more efficacious that waitlist or active control.	VR exposure therapy was highly effective in treating anxiety disorders.
			Significant improvements were observed in subjective distress, cognitive and behavioral measures, and psychophysiological measures.	Behavioral avoidance tests should be administered to assess the impact of treatment on anxiety-provoking situations and generalization to the real world.
			VR therapy was more effective than *in vivo* exposures.	
			Non-significant trend toward a dose-response relationship was observed between number of sessions and outcome measures.	


*CBT, cognitive behavioral Therapy; PTSD, post-traumatic stress disorder; VR, virtual reality.*

Whilst the existing literature on the use of VR therapy for phobias, panic disorder, and PTSD were promising, several limitations must be considered (Powers and Emmelkamp, 2008; Meyerbroker and Emmelkamp, 2010; Opris et al., 2012). Meyerbroker and Emmelkamp (2010) noted that VR as a therapeutic tool is difficult to assess as it is often combined with other techniques. This potentially masks any underlying benefits of VR therapy on the patient. Furthermore, most studies do not include behavioral avoidance tasks, which would help to determine how transferable the results are to the real world.

### Schizophrenia

As an assessment tool, VR offers the possibility of creating unique environments allowing researchers to better identify and understand specific areas of the brain commonly effected in schizophrenia. It is proposed that binding errors during the memory encoding process are responsible for the episodic memory impairments reported in schizophrenia (Waters et al., 2004). In this sense, VR is able to tease out areas of the brain responsible for binding impairments by providing specific situations or tasks for which patients have to perform. For example, a study by Ledoux et al. (2013) examined contextual binding in schizophrenia using fMRI during a navigation task in a virtual town (i.e., find the grocery store from the school). Their results showed significantly less activation among patients relative to controls in the left middle frontal gyrus, and right and left hippocampi. Ledoux et al. (2013) further suggested that the reduced activation was indicative of context and content not being appropriately linked, therefore affecting the formation of a cognitive map representation in the patient group and eliciting a contextual binding deficit.

As a rehabilitation tool, VR offers a unique potential to expose individuals to controlled rehabilitation environments and allow for interaction within a VE. Indeed, VEs may be perceived as less intimidating for patients as it allows for more gradual increase in task difficulty and may therefore enhance participation with rehabilitation (Rizzo et al., 1998). In particular, VR therapy has been explored as an alternative option to improve cognitive function (Marques et al., 2008) and vocational skills (Tsang and Man, 2013) in schizophrenic patients with some success. However, perhaps one of the most important roles of VR therapy may be to attenuate the deficit in social skills associated with schizophrenia. Traditionally, social skills training using role-play has been effective in remediating these deficits (Benton and Schroeder, 1990), however, role-playing of social skills training are limited in that they require appropriately matched groups, and may produce social anxiety, negative symptoms and poor insight. VR-based techniques offer an alternative to traditional role playing techniques by providing a computer-generated but realistic three-dimensional world and human-like avatars that can provide emotional stimuli. These VR-based techniques may be highly beneficial to re-train conversational skills (i.e., beginning a conversation, breaking silences, and differentiating facial expressions; Ku et al., 2007; Park et al., 2011).

While there is great potential for the role of VR in the treatment of schizophrenia, the evidence for its use remains contentious. Questions still remain if the effects of VR directly affects the condition itself, or perhaps the effects of VR may attenuate other psychiatric comorbidities such as anxiety or depression that may trigger visual or auditory hallucinations in sufferers of schizophrenia. As these are still early days for VR therapy in general, there is a need to determine the precise role of VR in treatment therapies for schizophrenia and its limitations.

## Future Outlook to the Most Promising Research Avenues - Complementing VR Therapy Using Non-Invasive Neuromodulation and Neuroimaging Technologies

This review so far has provided evidence for the use of VR therapy in various clinical populations as a standalone or adjunctive tool with mainstream neurorehabilitation treatment modalities. However, the question remains as to whether the beneficial effects of VR can be augmented via neuromodulation techniques such as tDCS, or if it is possible for a more targeted approach to monitor the effects of VR via non-invasive and portable neuroimaging methods such as fNIRS and EEG.

### Augmenting VR Therapy with tDCS

Transcranial direct current stimulation is an emerging non-invasive brain stimulation technique that uses low-intensity constant direct electrical currents to modulate the excitability of cortical neurons and related networks (Nitsche and Paulus, 2000; Lang et al., 2005). By placing either a positive anode or negative cathode electrode over the scalp of the head, tDCS is able to facilitate (anodal tDCS) or inhibit (cathodal tDCS) excitability of the underlying cortical neurons in a polarity-specific manner. Due to this robust neuromodulatory effect, tDCS has often been used in conjunction either before (offline) or during (online) rehabilitation therapy to improve motor and cognitive performance in healthy and clinical populations (for a recent reviews, see Coffman et al., 2014 and Floel, 2014).

In theory, the application of tDCS with VR therapy to augment neurorehabilitation appear complimentary. A study by Lee and Chun (2014) showed an improvement in stroke-specific clinical measures, manual muscle test and the Korean-modified Barthel Index in subacute stroke patients after 15 sessions of VR therapy with online cathodal tDCS to the unaffected motor cortex compared to sham. Kim et al. (2014) further demonstrated that the addition of online anodal tDCS to the affected motor cortex with VR therapy not only improved upper arm function, but also increased corticospinal excitability in subacute stroke patients.

In contrast to the aforementioned findings, mixed results were reported by Viana et al. (2014) that compared the effects of combining VR with offline anodal tDCS over the affected motor cortex of stroke patients, across 15 1-h VR therapy sessions. While the results showed no statistical differences in stroke-specific clinical measures (i.e., Fugl-Meyer assessment, Wolf motor assessment, and modified Ashworth scale) of upper arm function between patients receiving real tDCS compared to sham, it is important to note that more than 50% of participants receiving anodal tDCS and VR therapy had clinically significant improvements in wrist spasticity following treatment. Based on these limited combined VR and tDCS findings, it can be seen that performing tDCS during the VR therapy is a significant factor for enhancing the effects of VR therapy alone, which is also the case for combining tDCS with neurorehabilitation (Rothwell, 2012). Although combined VR and neuromodulation (tDCS) has been primarily applied in movement disorders, to the best of our knowledge, there are currently no known studies that have investigated this combination in cognitive and mood disorders. Thus therapists that are currently adopting the use of VR therapy in mental and mood disorders can potentially exploit the concurrent use of both VR and tDCS to augment therapy benefits above and beyond VR therapy alone.

It is likely that the combined effects of VR and tDCS is influenced by a combination of several factors, namely (1) general patient characteristics (e.g., brain region affected, and structural/functional reserve) and (2) tDCS parameters including electrode placement (affected or unaffected brain region), polarity (anodal or cathodal) and timing of tDCS application (online or offline). In such circumstances, where the efficacy of combined VR and tDCS interventions are both timing and location dependent (i.e., when and where to stimulate), a method of detecting and monitoring changes to neurophysiological function as patients receive treatment is crucial for optimizing intervention effects. In this regard, neuroimaging methods could be applied to monitor treatment VR progression, which will be discussed in the subsequent section.

### Monitoring VR Therapy with Neuroimaging

Neurophysiological changes associated with VR neurorehabilitation can be measured by non-invasive and portable neuroimaging techniques including fNIRS and/or EEG, to ascertain changes in cerebral hemodynamic responses or oscillatory brainwaves, respectively. In particular, the use of fNIRS as a tool to measure online cerebral hemodynamic responses during neurorehabilitation has received attention (for review see Irani et al., 2007; Ferrari and Quaresima, 2012). The use of fNIRS as a neuroimaging method relies on the principle of neurovascular coupling that measures the increase in regional cerebral blood flow (i.e., increase in oxygenated and decrease in deoxygenated hemoglobin) induced by neuronal activation, which is analogous to the blood-oxygenation-level-dependent responses measured by fMRI (Ferrari et al., 2004). Cortical activation measurements by fMRI and fNIRS techniques show highly correlated results in both motor and cognitive tasks (Huppert et al., 2006; Cui et al., 2011; Muthalib et al., 2013). While the application of fNIRS techniques is gaining popularity, EEG has long been used to measure online brain activity during a cognitive or motor task, and in various clinical populations (Bonanni et al., 2008; Gosselin et al., 2011). Of particular importance, EEG is used to detect changes in various brainwaves (i.e., Gamma, Alpha, Beta, Theta, and Delta) which are differentially affected by changes in mood (Huang and Lo, 2009), wakefulness (De Gennaro et al., 2001), neurological diseases (Bonanni et al., 2008), and brain injury (Gosselin et al., 2011). Both fNIRS and EEG have several advantages over fMRI, as they are portable, relatively inexpensive to use, and easy to operate with high temporal resolution. Furthermore, new generation systems are battery operated, wireless and further miniaturized to the size of a smartphone, ideal for ambulatory and untethered measurements consistent with a neuroergonomics approach (Ayaz et al., 2013; de Lissa et al., 2015).

As the use of fNIRS and EEG techniques in VR therapy is still relatively new, most studies to date have focused on healthy individuals (Bayliss and Ballard, 2000; Mingyu et al., 2005; Holper et al., 2010; Seraglia et al., 2011; Basso Moro et al., 2014), with the potential for more studies in clinical populations emerging as the popularity of these portable neuroimaging technologies increases. The current use of fNIRS and EEG in VR therapy has two proposed roles; (1) to monitor and provide augmented feedback regarding regions of cortical activation during therapy and (2) to use fNIRS or EEG as part of a BCI paradigm for therapy. In support of the first role, several studies have investigated the efficacy of fNIRS and EEG to record cortical hemodynamic and oscillatory changes during actual motor tasks and motor imagery in a VR environment. These studies demonstrated the efficacy of fNIRS and EEG to detect task-specific changes in cortical hemodynamics (Holper et al., 2010; Seraglia et al., 2011; Basso Moro et al., 2014) and oscillatory patterns (Bayliss and Ballard, 2000; Mingyu et al., 2005).

The ability of fNIRS and EEG to detect changes in these neurophysiological measures can provide both feedback on location and level of activation, for which clinicians and users can use to set intensity and progression of therapy. Furthermore, feedback on cortical activation can also be used to identify areas of hypo- or hyperactivity, which can be modulated using neuromodulatory techniques such as tDCS (see Prospective Integration of Neuromodulation-Neuroimaging with VR Therapy). In support of the second role, identifying cortical areas of activation, patterns and timing of cortical activation associated with various movements or mood states may also be recorded as classifiers for BCI training. Indeed, most BCI studies to date have employed the use of EEG as a measure for cortical activation for which to control a robotic limb or avatar in a VR environment (Lin et al., 2007; Formaggio et al., 2013, 2015). Although relatively new, there are also fNIRS-based BCI approaches demonstrating feasibility for future integration with VR and neurorehabilitation (Sitaram et al., 2007; Hong et al., 2015). Moreover, joint use of fNIRS- and EEG-based BCI approaches have also been demonstrated (Koo et al., 2015; Xuxian et al., 2015). This shows the potential to adopt fNIRS in a similar manner, whereby appropriate cortical hemodynamic responses can be classified to control a robotic or computer interface.

### Prospective Integration of Neuromodulation-Neuroimaging with VR Therapy

**FIGURE 2:**
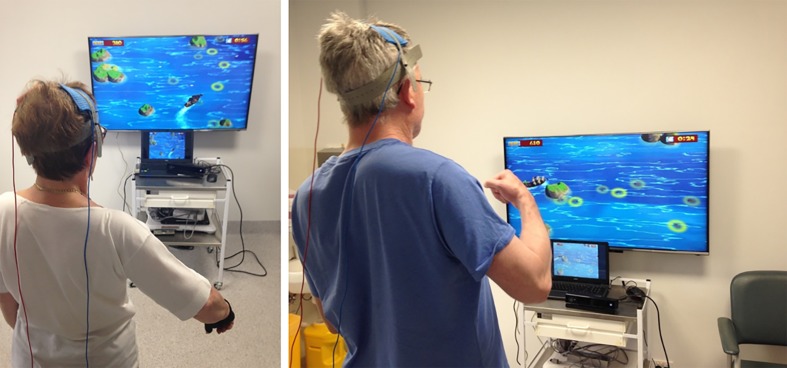
**Stroke participants engaged in VR therapy using an X-Box Kinect motion capture system while receiving tDCS**.

**FIGURE 3:**
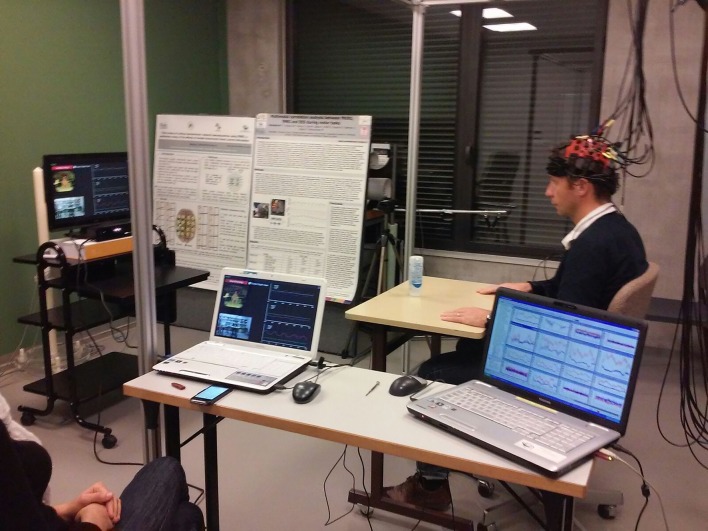
**The use of a semi-immersive VR environment and fNIRS system**.

In the last 5 years, new research suggests that the combination of VR therapy with neuromodulation and neuroimaging techniques may help to improve the effects and delivery of VR therapies. Neuromodulation techniques such as tDCS (**Figure 2**) and neuroimaging methods such as fNIRS (**Figure 3**) and EEG have already been combined and have shown some success (Ang et al., 2015; Dutta et al., 2015; Muthalib et al., 2016). While the use of these techniques in combination with VR is still in its infancy, the available evidence suggests highly complementary effects when combining neuromodulation and neuroimaging with VR therapy.

As both neuroimaging (fNIRS/EEG) and neuromodulation (tDCS) techniques have complimentary capabilities, and they both can be built as wearable and wireless systems, integration of the two presents as a natural avenue for applications in natural environments and real world settings (McKendrick et al., 2015). One potential use is enhancing BCI applications. In its most general form, BCI provides a route for neural output that does not involve the neuromuscular system (Wolpaw et al., 2002). Almost all current non-invasive BCI systems are read-only, that is, brain signals are read directly to the system (via neuroimaging) and the system can provide an output or feedback that relies on sensory input mechanisms and peripheral nervous system to reach back to the brain. A more direct BCI could eliminate the need for sensory input with the use of neuromodulation, and hence provide feedback directly to the brain for a read-write BCI.

Integration of VR therapy for such a read/write BCI hold potential for enhanced or accelerated therapeutical processes in neurorehabilitation. A study by Ang et al. (2015) was one of the first studies that combined VR with both tDCS and EEG to investigate the additive effect of offline anodal tDCS on BCI haptic training with chronic stroke patients. Although this study reported no difference in the Fugl-Meyer assessment and block-and-box test between groups that received real tDCS prior to BCI training, compared to sham, the study did demonstrate an increased state of EEG mu rhythm suppression in the real tDCS group. Thus indicating improved neurophysiological responses in motor preparation. Future studies are necessary to determine the significance of these neurophysiological improvements to clinical outcomes.

Neuroimaging guided tDCS and VR therapy can be applied to neurorehabilitation in general. The optimal location for applying tDCS electrodes to modulate a target cortical region and connected networks is a debated point in the field. Modeling studies of current flow between the tDCS electrodes have provided some guidance to the placement of electrodes to stimulate a specific brain region; however, whether these models predict the actual current flow and polarization of the targeted brain networks is not known. Simultaneous tDCS-neuroimaging could provide a solution to confirming the modeling predictions and/or to guide the direction of current flow between multi-electrode tDCS montages (Ruffini et al., 2014). For example, a motor task could be used to activate a broad network of cortical regions of interest that can be measured using fNIRS/EEG neuroimaging. Once the locations of the tDCS electrodes have been determined using neuroimaging guidance, neuroimaging and tDCS could be simultaneously or independently used to guide and modulate VR therapy. In this scenario, neuroimaging during VR tasks can be used to adapt the intensity based on the level of activation, such as the attentional hub of the dorsolateral prefrontal cortex (DLPFC). The level of DLPFC activation during the initial task would be expected to be high due to the novelty of the activity, however, as the activity progresses the learning of task requirements become automatic and performance improves, less attentional resources would be required, and the level of DLPFC would be expected to decrease (Ayaz et al., 2012; McKendrick et al., 2014). In such a scenario, the VR task can be adapted online to modulate the intensity level and maintain optimal DLPFC activation. Also, if the levels of DLPFC activation remain at high levels and/or performance is stagnant, then tDCS could potentially be applied online to upregulate neuronal networks required to perform the task. Preliminary evidence using a modeling approach to locate tDCS electrodes was provided by McKendrick et al. (2015) using a spatial memory task with concurrent fNIRS and tDCS. They showed that when task performance declined rapidly following baseline, the application of tDCS almost immediately eliminated the performance decrement. Furthermore, they showed that tDCS can modulate the neural activity of specific brain regions near the site of stimulation. However, they cautioned that current models and protocols for determining tDCS montages are lacking, due to complex interactions between stimulation montage, task performance and underlying hemodynamics that are not fully understood. Therefore, additional joint tDCS and fNIRS/EEG studies are required to further unravel these complexities and to better define the pattern of cortical excitation induced by tDCS during the performance of cognitive and motor tasks.

## Current Limitations of VR Therapy

While using a VE offers many unique advantages to traditional treatment and neurorehabilitation approaches, limitations to their efficacy and practicality must be acknowledged. Firstly, larger clinical studies are required to establish the efficacy of using VR in physical and cognitive rehabilitation in different clinical populations. Much of the existing literature report mixed findings from small sample sizes, and often lack appropriate control comparisons (see **Tables 1** and **2**). Secondly, there is little information on the transfer of the training effects of VR into the corresponding physical environment in general, and the VR training parameters associated with optimal transfer to real-world functional improvements are yet to be elucidated. Thirdly, in many clinical populations it is unclear whether advantages of VR over real-world training exist, and if so, precisely what these advantages are (see **Tables 1** and **2** for study limitations). Furthermore, because this literature is extremely vulnerable to selective reporting and Type-I statistical error, there is an inherent bias of publishing results that show correlation between rehabilitation improvements and the application of VR. Therefore to potentially limit any bias in future studies, it may be useful for future studies to adopt a double-blinded protocol for the evaluation of the effectiveness of the use of VR. Lastly, it is important to investigate any unique rehabilitative effects of VR that may be exploited, or whether the benefits of VR can be attributed to the enjoyment of gaming platforms associated with VR themselves (i.e., VR therapies may only present as more effective because they engage and motivate participants throughout their training session, providing increased adherence). While limitations in VR technology exist, the potential for favorable neuroplasticity afforded by such technology undoubtedly warrants further investigation.

## Conclusion

In summary, this review has discussed the strengths and limitations for the use of VR therapies in motor and mental health neurorehabilitation. The current evidence suggest that a combination of VR and conventional therapies are safe and likely to be more efficacious compared to just traditional or VR therapy alone. However, it is not known if the use of VR therapies can lead to cost-saving benefits (i.e., reduced financial and manpower cost) or even if current commercial or customized systems will be applicable by patients that are living within the community. More importantly, there is a need to elucidate the aspects of VR that are most effective for rehabilitation. While this is not apparent in the current review, future studies should attempt to systematically determine the role of self-projection, sensory feedback or motivation on rehabilitation in relation to specific diseases or impairments. Furthermore, we have discussed the potential for VR therapy to be complemented by other forms of technologies such as neuromodulation (tDCS) and neuroimaging (fNIRS/EEG) in order to augment training benefits of VR, and provide a more targeted approach to neurorehabilitation. Large-scale longitudinal studies will also be required to determine the effects of VR therapy (in combination with tDCS/fNIRS/EEG) and the translation of VR therapy in a non-clinical environment (i.e., home setting).

## Author Contributions

All authors listed, have made substantial, direct and intellectual contribution to the work, and approved it for publication.

## Conflict of Interest Statement

The authors declare that the research was conducted in the absence of any commercial or financial relationships that could be construed as a potential conflict of interest.

## Abbreviations

BCI: brain computer interface
CP: cerebral palsy
EEG: electroencephalography
fMRI: functional magnetic resonance imaging
fNIRS: functional near-infrared spectroscopy
PD: Parkinson’s disease
PTSD: post-traumatic stress disorder
tDCS: transcranial direct current stimulation
VE: virtual environment
VR: virtual reality
