# Corrigendum: Does a Combination of Virtual Reality, Neuromodulation and Neuroimaging Provide a Comprehensive Platform for Neurorehabilitation? – A Narrative Review of the Literature

**DOI:** 10.3389/fnhum.2017.00053

**Published:** 2017-02-03

**Authors:** Wei-Peng Teo, Makii Muthalib, Sami Yamin, Ashlee M. Hendy, Kelly Bramstedt, Eleftheria Kotsopoulos, Stephane Perrey, Hasan Ayaz

**Affiliations:** ^1^Institute for Physical Activity and Nutrition (IPAN), Deakin UniversityBurwood, VIC, Australia; ^2^EuroMov, University of MontpellierMontpellier, France; ^3^Cognitive Neuroscience Unit, Deakin UniversityBurwood, VIC, Australia; ^4^Liminal Pty Ltd.Melbourne, VIC, Australia; ^5^Adult Mental Health, Monash HealthDandenong, VIC, Australia; ^6^School of Exercise and Nutrition Sciences, Deakin UniversityBurwood, VIC, Australia; ^7^Aged Persons Mental Health Service, Monash HealthCheltenham, VIC, Australia; ^8^School of Biomedical Engineering, Science and Health Systems, Drexel UniversityPhiladelphia, PA, USA; ^9^Department of Family and Community Health, University of PennsylvaniaPhiladelphia, PA, USA; ^10^The Division of General Pediatrics, Children's Hospital of PhiladelphiaPhiladelphia, PA, USA

**Keywords:** neurorehabilitation, neuroplasticity, tDCS, fNIRS, EEG, virtual reality therapy

In the original article, there was a mistake in the legend for Figure 2 as published. The correct legend appears below. The authors apologize for this error and state that this does not change the scientific conclusions of the article in any way.

**Figure 2. Stroke participants engaged in VR therapy using an X-Box Kinect motion capture system, MediMoov by NaturalPad, while receiving tDCS**.

## Conflict of interest statement

The authors declare that the research was conducted in the absence of any commercial or financial relationships that could be construed as a potential conflict of interest.

